# Large-Scale Measurements of Physical Activity With Wearable Devices: An International Perspective

**DOI:** 10.1093/geroni/igaa057.2755

**Published:** 2020-12-16

**Authors:** Jacek Urbanek, Jennifer Schrack, David Roth

## Abstract

In recent years the popularity and application of both research- and consumer-grade wearable physical (PA) activity monitors have witnessed substantial growth in large observational studies and clinical trials. For example, the NHANES and UKBiobank, have collected accelerometry data on thousands of participants contributing to the reputation of wearable technology overall as well as in aging-oriented research. As a result, more aging-focused studies including the Baltimore Longitudinal Study of Aging, Maastricht Study, Finnish Retirement and Aging study, and the National Health and Aging Trends Study, along with clinical trials have introduced accelerometry protocols into their design. The symposium focuses on challenges in the implementation of the objective measurements of PA into large studies on older adults. We will discuss the design of successful projects held and/or completed in the United States and Europe including: (1) types of devices, (2) size of datasets, (3) steps necessary for the successful device implementation, (4) data management and (5) statistical analyses. We will also present primary, PA-related findings in each study, together with funded or planned follow-up work. Collectively, these presentations will improve understanding of the technology and effort necessary for the successful application of objective PA monitoring and the resulting data analysis, providing a better context for investigators in the field of aging who want to introduce wearable devices into existing and upcoming research. The discussion will focus on the future of these technologies in the context of geriatric medicine and gerontology and the consequent steps essential for their best utilization and further expansion.

